# Multiple Implants Do Not Aggravate the Tissue Reaction in Rat Brain

**DOI:** 10.1371/journal.pone.0047509

**Published:** 2012-10-16

**Authors:** Gustav Lind, Lina Gällentoft, Nils Danielsen, Jens Schouenborg, Lina M. E. Pettersson

**Affiliations:** Department of Experimental Medical Sciences, Neuronano Research Center, Medical Faculty, Lund University, Lund, Sweden; Tokyo Medical and Dental University, Japan

## Abstract

Chronically implanted microelectrodes are an invaluable tool for neuroscientific research, allowing long term recordings in awake and behaving animals. It is known that all such electrodes will evoke a tissue reaction affected by its’ size, shape, surface structure, fixation mode and implantation method. However, the possible correlation between tissue reactions and the number of implanted electrodes is not clear. We implanted multiple wire bundles into the brain of rats and studied the correlation between the astrocytic and microglial reaction and the positioning of the electrode in relation to surrounding electrodes. We found that an electrode implanted in the middle of a row of implants is surrounded by a significantly smaller astrocytic scar than single ones. This possible interaction was only seen between implants within the same hemisphere, no interaction with the contralateral hemisphere was found. More importantly, we found no aggravation of tissue reactions as a result of a larger number of implants. These results highlight the possibility of implanting multiple electrodes without aggravating the glial scar surrounding each implant.

## Introduction

Multielectrode arrays, such as chronically implanted electrodes for stimulation or recording within the central nervous system (CNS), show great promise as research tools and diagnostic and therapeutic devices in years to come [1], [2], [3]. To achieve this, multiple multielectrode arrays have to be functional inside the CNS for years or decades without causing significant damage to the surrounding tissue. However, all types of electrodes available today show deteriorating recording capabilities over time [4], [5], [6]. This is suggested to be, at least partly, due to the tissue reactions surrounding the electrodes that over time will increase the impedance of the electrodes. This may ultimately insulate the recording surfaces and thus prevent recording of electrical signals or forcing stimulation parameters to be altered [7], [8], [9], [10], [11]. The tissue reactions surrounding different types of neural implants have been extensively studied with regards to size [12], [13], shape [10], [14], [15], [16], surface structure and material [17], [18], [19], [20], fixation mode [13], [21] and implantation method [14], [22]. However, to the best of our knowledge, interactions between the tissue reactions to multiple electrodes implanted in the brain have not been studied. This is a key question to be answered since the potential additive effect of multiple glial scars could affect both the quality of recordings from implanted electrodes and the validity of the neural signals recorded.

The aim of this study was to evaluate the effect of the number of implants on the glial scar, defined in this study as the accumulation of reactive astrocytes and activated microglia, surrounding each single implant. Two different aspects of this problem were investigated. First, we examined whether the tissue reaction to an electrode was affected by implantation of other electrodes in its close vicinity, i.e. if glial scars interact within a hemisphere. Second, we examined whether the tissue reactions to an electrode is affected by the presence of contralateral implants, i.e. if glial scars interact between hemispheres. We chose to focus on the astrocytic and microglial reactions, which are main components of the glial scar, and the most commonly investigated [4], [10], [12], [13], [15], [20], [21], [23], [24], [25]. The astrocytic reaction is monitored by measuring immunoreactivity to GFAP, an intermediate filament protein expressed in all astrocytes but highly up regulated in reactive astrocytes in response to an injury. The astrocytes are also the constituent of the *glia limitans* layer surrounding implanted materials which delimit the normal neural tissue from the damaged tissue and implanted materials. To monitor the microglial reaction we measure immunoreactivity to ED1, a cellular surface protein expressed exclusively on cells of monocytic lineage, in the brain primarily microglial cells, when activated by an injury. These cells are mainly responsible for phagocytosis of damaged tissue and foreign material and are thus a good measure of the damage caused by an implant. In addition, activation of these glial components has been claimed to correlate with alterations in impedance of implanted electrodes [9], [11].

After six weeks, the astrocytic scar surrounding the middle out of five implants was significantly smaller compared to the single contralateral implant, suggesting that an intrahemispheric interaction might be taking place, reducing the astrocytic response around the central implant. However, we did not find any evidence of interactions between hemispheres. Furthermore, we did not find any difference between microglial reactions in the different groups. Most importantly however, the large number of implants did not seem to aggravate the reaction to any of the implants, indicating a possibility of implanting multiple electrodes at diverse locations in the brain.

## Methods

### Animals, Anaesthesia and Ethics Statement

All procedures in this study were approved in advance by the Malmö/Lund Animal Ethics Committee on Animal Experiments. Implantations were made in female Sprague-Dawley rats (n = 23) (Taconic, Denmark), weighing 200–250 g. Animal handling and anaesthetic procedures are described elsewhere [26]. In brief, animals were anaesthetized with intraperitoneal injections of fentanyl (0.3 mg/kg body weight) and Domitor vet (medetomidin hydrochloride, 0.3 mg/kg body weight). After surgery, the animals received subcutaneous injections of an antidote to the anaesthesia (Antisedan, atipamezole hydrochloride, 0.5 mg/kg body weight) as well as Temgesic (buprenorphine, 50 µg/kg body weight) to reduce postoperative pain.

### Implants

**Figure 1:**
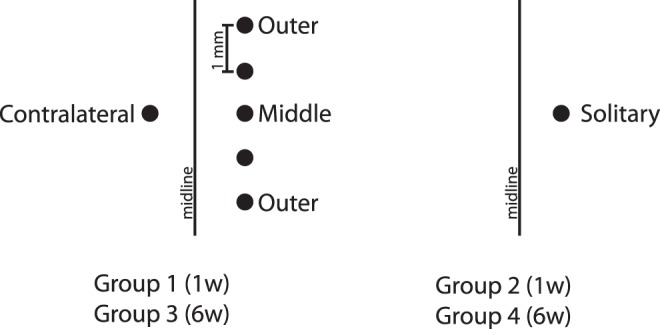
Schematic overview of implant locations in the different study groups. Dorsal view of the cerebral cortex with implant locations indicated by black dots. The implant in the left hemisphere of Group 1 & 3 is referred to as the contralateral implant; the implants in the right hemisphere in group 1 & 3 are referred to as middle and outer implants respectively; the implant in the right hemisphere of group 2 & 4 is referred to as the solitary implant and has no contralateral counterpart. Distances between implants in the right hemisphere of group 1 & 3 are 1 mm. Unnamed implants are not analyzed.

The implants in this study were identical to the gelatine embedded wire bundles used in a previous study in our laboratory [25]. Implants consist of a wire bundle of 32 tungsten wires with a diameter of 7.5 µm and an insulation layer of 3 µm polyimide,moulded into a gelatine needle (gelatine type B, VWR BDH, Sweden) resulting in a final diameter of 300 µm. The gelatine is intended to give stability to the highly flexible wires while penetrating the meninges. It dissolves during, or soon after, implantation leaving only the wire bundle in place in the cortex. The wire bundle have an approximate diameter of 180 µm. Animals were kept for one or six weeks and were divided into the following experimental groups; 1) killed after one week, implanted with five wire bundles in the left hemisphere with 1 mm between each bundle, and one wire bundle in the right hemisphere (n = 6); 2) killed after one week, implanted with one wire bundle in the left hemisphere, and no implant in the right hemisphere (n = 6); 3) killed after six weeks, implanted with five wire bundles in the left hemisphere, with 1 mm between each bundle, and one wire bundle in the right hemisphere (n = 6); 4) killed after six weeks, implanted with one wire bundle in the left hemisphere, and no implants in the right hemisphere (n = 5). A schematic overview of the groups is presented in Figure 1.

### Surgery and Implantation Procedure

The animal was attached to a stereotactic frame (KOPF instruments, USA) under anaesthesia prior to surgical procedures. Small craniotomies (1 mm^2^) were made at the single bundle implantation site while one large craniotomy (6×1 mm) was made at the five bundle implantation site. The dura mater was incised and deflected. Implants were attached to a hydraulic micromanipulator (KOPF instruments, USA) using gelatine. Implantations were made one bundle at a time at a speed of 10 µm/s, to a depth of 2 mm. Once the target depth was reached, the gelatine attaching the implant to the micromanipulator was flushed with saline solution until dissolved, releasing the implant. This method is designed to be able to release the implants without moving them while inside the brain. The implants were left untethered without any attachment to the skull or each other. This ensures that the electrodes move along with the brain, and do not translate movements between the brain and the skull which is thought to be one of the major causes of chronic reactions to neural implants. The skin was closed using surgical clips and the animals were monitored during awakening.

### Histology

The animals were killed by an i.p. overdose of pentobarbital and were transcardially perfused with 200 ml of ice-cold 0.1 M phosphate buffered saline (PBS) followed by 150 ml of ice-cold 4% paraformaldehyde in 0.1 M phosphate buffer, pH 7.4. The brains were dissected and immersed in 4% paraformaldehyde overnight. The brains were then cryoprotected in 25% sucrose until they were no longer able to float and were cryosectioned horizontally using a cryostat (Microm, Germany) in increments of 10 µm onto Super Frost ® plus slides (Menzel-Gläser, Germany). After blocking in goat serum to prevent unspecific binding, the sections were incubated with primary antibodies; rabbit anti-glial fibrillary acidic protein (GFAP, an astrocytic cytoskeleton protein; 1∶5000, Cat. Nr. Z0334, Dako, Denmark) and mouse anti-CD68 (ED1, a marker for activated microglial cells, 1∶250, Cat. Nr. MCA341R, AbD Serotec, UK) at room temperature overnight. The specificities of the antibodies have been tested elsewhere (GFAP by the manufacturer and ED1 by Bao et al. [27]) by Western Blot (two-dimensional quantitative immunoelectrophoresis or SDS-PAGE respectively). Both antibodies show a single precipitate with roughly the appropriate molecular weight when tested on brain or spinal cord extract, indicating good specificity for the targeted antigen. Thereafter, sections were rinsed three times in PBS followed by incubation in secondary antibodies; goat anti-rabbit Alexa594 (1∶500, Invitrogen, USA) and goat anti-mouse Alexa488 (1∶500, Invitrogen, USA) for 2 hours in the dark at room temperature. Sections were rinsed three times in PBS and coverslipped using PVA/DABCO (Fluka/Sigma-Aldrich, Switzerland).

**Figure 2:**
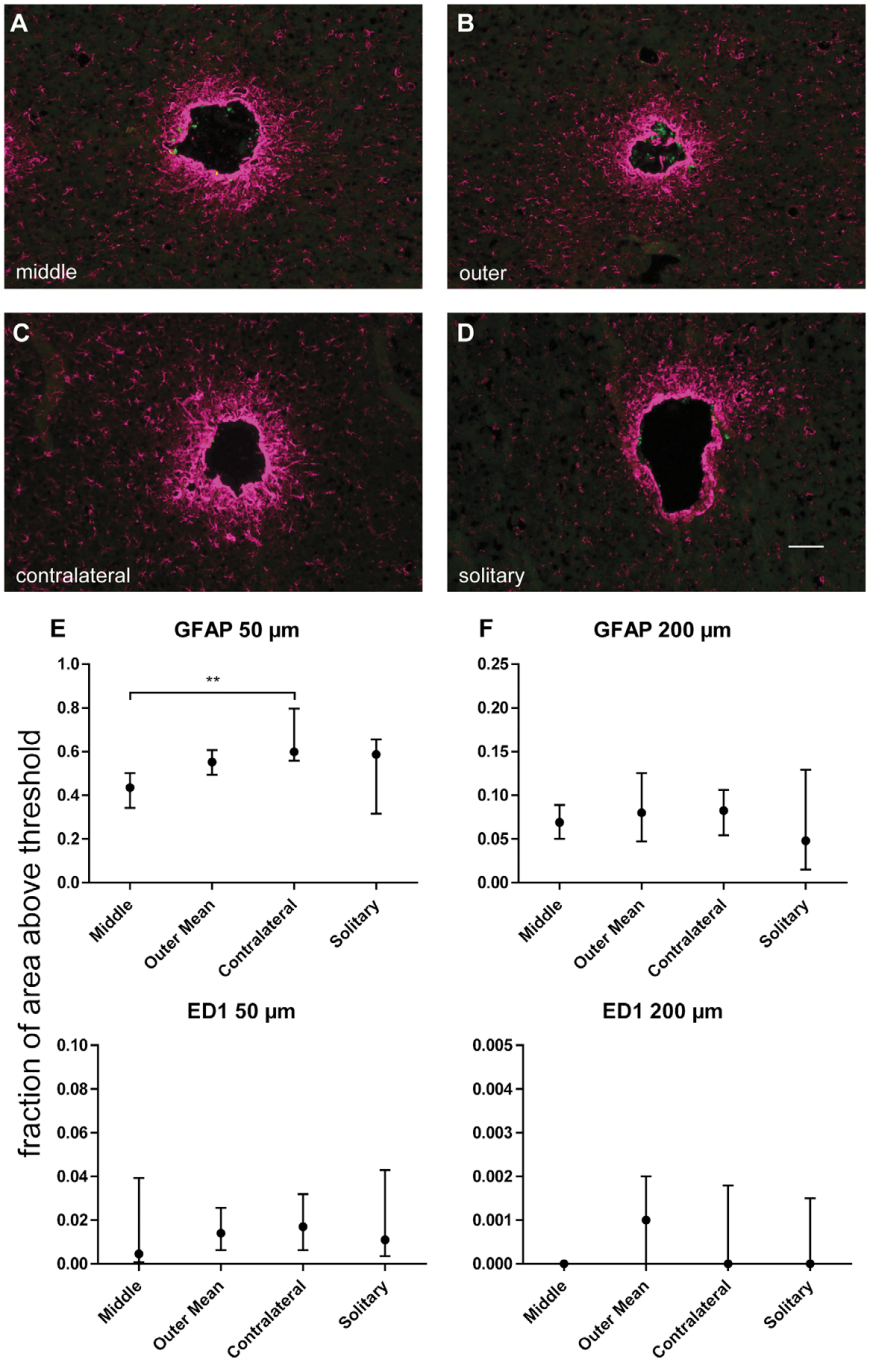
Glial reactions surrounding implants after six weeks. Example pictures of GFAP (magenta) & ED1 (green) staining from middle (A), outer (B), contralateral (C) & solitary (D) implant locations after six weeks. Scale bar 100 µm. Quantifications of GFAP & ED1 staining after six weeks in 0–50 µm ROI (E) & 50–200 µm ROI (F). X-axes show different implant locations. Y-axes show the fraction of area in each ROI that is above the set threshold. The astrocytic scar surrounding the middle implant was significantly smaller than surrounding the contralateral implant in the inner ROI (p<0.01, K-W test with Dunn’s post hoc test). No difference was found for ED1 staining between any of the groups, n = 6 for all groups except “solitary” where n = 5.

**Figure 3:**
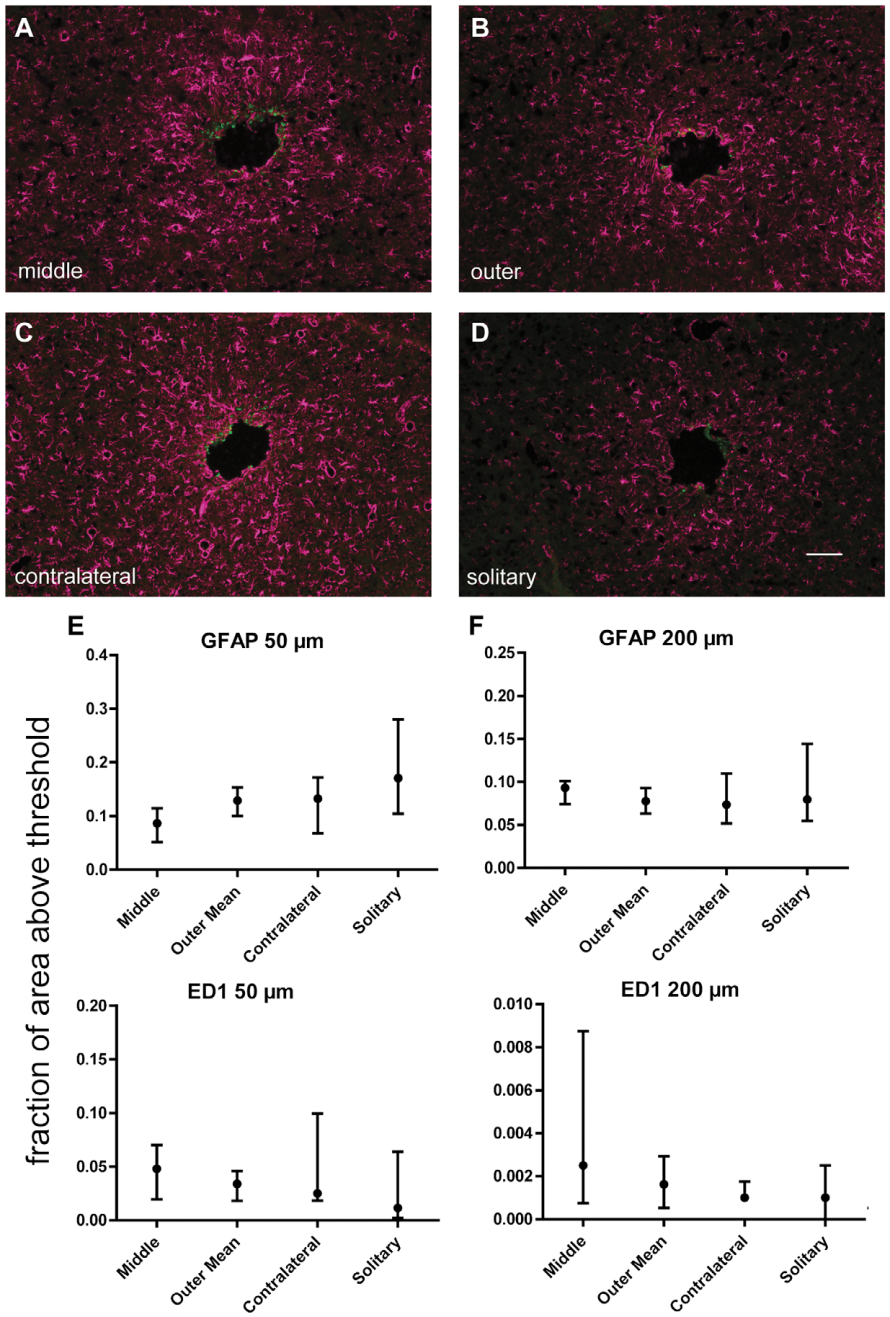
Glial reactions surrounding implants after one week. Example pictures of GFAP (magenta) & ED1 (green) staining from middle (A), outer (B), contralateral (C) & solitary (D) implant locations after one week. Scale bar 100 µm. Quantifications of GFAP & ED1 staining after one week in 0–50 µm ROI (E) & 50–200 µm ROI (F). Circles & error bars signify median values & interquartile ranges. X-axes show different implant locations. Y-axes show the fraction of area in each ROI that is above the set threshold. No statistically significant differences between the tissue reactions at the different implant locations was found in any of these groups, n = 6 for all groups.

### Image Acquisition & Analysis

All histological images were obtained using a DS-2MV Digital camera (Nikon, Japan) mounted on a Nikon eclipse 80i microscope with a 10x objective. Image capture and analysis were performed using the NIS-Elements 3.1 software (Nikon Instruments). Images from the middle of the shaft of each bundle track, at an approximate depth of 1 mm below brain surface, were captured from the middle and outer bundles, their contralateral counterpart, as well as the solitary bundles (see Figure 1 for explanation). Regions of interest (ROIs) were set at 0–50 µm and 50–200 µm distance from the rim of the artefact left by the wire bundles. The histological quantification method has previously been described in detail [13], [25]. In brief, thresholds were set for each individual image and for each marker at a fixed multiplier of the mean background intensity. The fraction of the area in each ROI above this threshold was calculated. Intensity thresholds were set at six times the background intensity for GFAP immunofluorescence and at five times for ED1 immunofluorescence.

### Statistical Analyses

Non-parametric statistics, the Kruskal-Wallis and the Dunn’s post hoc test for selected pairs were used in this study. Comparisons were made between i) the middle implant and a mean of the two outer implants; ii) the middle implant and the contralateral implant; and iii) between the contralateral implant and the solitary implant (see figure 1 for explanation), for each ROI and time point. All values are presented as median values with indication of the interquartile range and p-values of <0.05 were considered significant. All analyses were performed using the GraphPad Prism 5.03 software (GraphPad Software Inc., USA).

## Results

### Interactions within a Hemisphere

To evaluate whether any interactions within a hemisphere were present we compared the tissue reactions towards the middle implant to that towards the contralateral implant and the outer implants (Figure 1). We found significantly less immunofluorescence for GFAP at the middle implant in the innermost ROI (0–50 µm) after six weeks when compared to the contralateral implant (Figure 2). This suggests that a large number of implants in one hemisphere do not aggravate the tissue reactions to each implant. Notably, the middle scar exhibited less GFAP staining than both the contralateral scar and the outer scars in every animal (in the inner ROI at the six week time point), even if the difference between the middle and outer scars was not statistically significant. Furthermore, there was a similar tendency after one week, where the contralateral implant exhibited higher GFAP levels than the middle implant in the inner ROI in all animals except one, and the outer implant exhibited higher levels than the middle implant in all animals (Figure 3). These differences were not statistically significant. No differences or tendencies to differences were seen between any of the groups when analysed with regards to ED1-staining (Figures 2, 3).

### Interactions between Hemispheres

Potential interactions between hemispheres were examined by comparing tissue reactions to the contralateral implant to those of the solitary implant (Figure 1). No statistically significant differences were found between these groups, neither for astrocytic nor microglial reactions, and the median values were similar in all groups (Figures 2, 3). This indicates that the five implants in one hemisphere do not affect the astrocytic reactions to the contralateral implant.

## Discussion

Concerns, regarding an adverse additive effect on tissue reactions after implantation of multiple electrodes into the brain, have been raised. This question is central to chronic electrophysiology since an escalating additive reaction could preclude the possibility of recording valid physiological signals from diverse brain areas. We wanted to address this issue and also investigate possible contralateral effects in response to implantation. Interestingly, our results indicate that glial reactions to chronic neural electrodes implanted in close vicinity of each other are not more severe than those to single electrodes. The significant difference seen between middle and contralateral implant in the six week group might even suggest that a larger number of implants slightly reduce the tissue reactions to each implant, but further studies would be needed to provide a definite answer to this. Most importantly, our findings indicate that it is possible to implant multiple electrode bundles without aggravating the glial scar surrounding each implant. This enables implantation of functional neural interfaces consisting of a number of electrodes implanted at different sites in the brain, which gives the opportunity to study how different remote brain areas interact with each other without the potential confounding factor of an increased glial scar.

The mechanism behind the decreased astrocytic scar formation around the central implant is not directly explored in this study, and can thus only be speculated on. It seems, however, that we have discovered an interesting feature of the development of reactive astrocytosis. The most intuitive reason for a difference in glial scar formation between two implants would be that there is a difference in the damage caused by these implants. In this study, the implants are identical, and the implantation procedures are identical, the only difference is the positioning of the electrodes. The only way we can envision that this would affect the damage caused by the electrodes is if the movements of the outer electrodes in relation to the brain are larger than of the middle ones, i.e. if there were to be a stabilizing effect. However, the electrodes in this study are free-floating, with no attachment to the skull or each other. Thus, the movements between the electrodes and the tissue should be minimal, and not likely to cause any significant differences between the different implants. Furthermore, the fact that no differences were seen between the groups after staining for ED1 also indicates that there is no difference in the actual damage caused by the different implants.

It should be pointed out that the implanted free-floating wire bundles are not identical to any functioning electrodes available today which require a solid connection to a connector on the skull. Our wire bundles are not attached to anything except the brain, which means that they are not affected by the movements of the skull in relation to the brain. Hence, if we had used electrodes tethered to the skull, the hemisphere implanted with a large number of electrodes would have been more stabilized in relation to the skull than the hemisphere with only one implant. Thus the movements between the electrodes and the brain would differ, as well as the resulting tissue damage caused by the relative movements. By using free-floating electrodes we hope to eliminate such movements, isolating the effect of the number of implant on the tissue reaction, which is the focal point of this study. Furthermore, even if a solid lead to the skull is a requisite for intracortical electrodes today, the development of implantable light weight telemetry units can be foreseen to provide means for using completely free-floating electrodes in electrophysiology in the near future [28].

If the damage caused by each implant is virtually identical, the difference in astrocytic scarring that we still see is most likely explained by some innate property of the astrocytes being recruited to the scar. Two main theories regarding how reactive astrocytes are recruited to an injury have been described [29], [30], [31]. Astrocytes may be recruited from local resident quiescent astrocytes that differentiate into reactive astrocytes and proliferate [30], [32], [33], or from distal precursor cells, for instance from the subventricular zone [34], [35].

If cells are being recruited from a distal source this might be mediated via chemotaxis as astrocytes are known to express chemokine receptors [36]. The presence of a larger chemotactical gradient in the middle of a group of implants, where all the implants may additively contribute to the gradient, than at the edges, is likely. In this case the middle implant would be able to recruit a larger proportion of astrocytes than the outer ones. However, if the astrocytes are competitively recruited from a local pool of quiescent astrocytes, the outer implants would have access to a larger pool of astrocytes compared to the middle ones who will have to compete with neighboring implants. In this scenario, the middle implant would exhibit a smaller scar than the outer ones, consistent with what we found in the present study. It should be pointed out that the proliferation of astrocytes is likely to contribute more to the amount of scarring than the initial number of recruited cells. Still, a difference in the initial number of astrocytes recruited could lead to a difference in the scar formation.

The point should also be raised that even if research regarding biocompatibility of multielectrode arrays today aim at reducing the reaction to implantation, the fact remains that reactive gliosis also has a very important defensive function. Indeed, numerous studies have shown detrimental effects of deletion of genes central to the process of reactive gliosis, such as absence of a normal glial scar, increased edema surrounding the injury, more extensive damage to the blood brain barrier and a significantly increased area of inflammation after injury [37], [38], [39], [40]. Thus, while reactive gliosis has detrimental effects on neuronal regeneration in the chronic phase, it is essential to limit the extent of a brain injury in the acute phase [37], [40], [41], [42]. The reduction of reactive gliosis should perhaps only be seen as positive when correlated with a reduced damage to the tissue, and therefore a reduced need for defensive and repair mechanisms. In our study, the inflicted tissue damage is the same at all implantation sites, and therefore also the need for protective mechanisms. Thus, if the tissue response is not able to keep up with the tissue damage, a reduced tissue reaction should perhaps even be considered to be negative, actually increasing the vulnerability of the neural tissue. Further long term studies are needed to investigate a possible cut off when the number of implants might be too large for the tissue response to handle.

In conclusion, our results suggest that it is possible to implant electrodes in multiple brain sites without aggravating the tissue response, thus providing validity to large-scale electrophysiological recordings from multiple chronically implanted electrodes. Our finding that the middle implant exhibits the least amount of reactive astrocytosis might suggest that the astrocytes are recruited to the injury site in a competitive manner from a local pool rather than an additive manner from distal migrating cells, but this mechanism is not investigated in this study and further studies would be required to shed light on the subject.

## References

[1] Awan NR, Lozano A, Hamani C (2009) Deep brain stimulation: current and future perspectives. Neurosurgical focus 27: E2. 10.3171/2009.4.FOCUS0982 19569890

[2] Daly JJ, Wolpaw JR (2008) Brain-computer interfaces in neurological rehabilitation. Lancet Neurol 7: 1032–1043. 18835541 10.1016/S1474-4422(08)70223-0

[3] Kipke DR, Shain W, Buzsaki G, Fetz E, Henderson JM, et al (2008) Advanced neurotechnologies for chronic neural interfaces: new horizons and clinical opportunities. J Neurosci 28: 11830–11838. 19005048 10.1523/JNEUROSCI.3879-08.2008 PMC3844837

[4] Polikov VS, Tresco PA, Reichert WM (2005) Response of brain tissue to chronically implanted neural electrodes. J Neurosci Methods 148: 1–18. 16198003 10.1016/j.jneumeth.2005.08.015

[5] Rousche PJ, Normann RA (1998) Chronic recording capability of the Utah Intracortical Electrode Array in cat sensory cortex. J Neurosci Methods 82: 1–15. 10223510 10.1016/s0165-0270(98)00031-4

[6] Williams JC, Rennaker RL, Kipke DR (1999) Long-term neural recording characteristics of wire microelectrode arrays implanted in cerebral cortex. Brain Res Brain Res Protoc 4: 303–313. 10592339 10.1016/s1385-299x(99)00034-3

[7] Biran R, Martin DC, Tresco PA (2005) Neuronal cell loss accompanies the brain tissue response to chronically implanted silicon microelectrode arrays. Exp Neurol 195: 115–126. 16045910 10.1016/j.expneurol.2005.04.020

[8] Krack P, Fraix V, Mendes A, Benabid AL, Pollak P (2002) Postoperative management of subthalamic nucleus stimulation for Parkinson’s disease. Movement disorders : official journal of the Movement Disorder Society 17 Suppl 3S188–197. 11948776 10.1002/mds.10163

[9] McConnell GC, Butera RJ, Bellamkonda RV (2009) Bioimpedance modeling to monitor astrocytic response to chronically implanted electrodes. J Neural Eng 6: 055005. 19721187 10.1088/1741-2560/6/5/055005

[10] Szarowski DH, Andersen MD, Retterer S, Spence AJ, Isaacson M, et al (2003) Brain responses to micro-machined silicon devices. Brain Res 983: 23–35. 12914963 10.1016/s0006-8993(03)03023-3

[11] Williams JC, Hippensteel JA, Dilgen J, Shain W, Kipke DR (2007) Complex impedance spectroscopy for monitoring tissue responses to inserted neural implants. Journal of Neural Engineering 4: 410–423. 18057508 10.1088/1741-2560/4/4/007

[12] Stice P, Gilletti A, Panitch A, Muthuswamy J (2007) Thin microelectrodes reduce GFAP expression in the implant site in rodent somatosensory cortex. Journal of Neural Engineering 4: 42–53. 17409479 10.1088/1741-2560/4/2/005

[13] Thelin J, Jorntell H, Psouni E, Garwicz M, Schouenborg J, et al (2011) Implant size and fixation mode strongly influence tissue reactions in the CNS. PLoS One 6: e16267. 21298109 10.1371/journal.pone.0016267 PMC3027655

[14] Edell DJ, Toi VV, McNeil VM, Clark LD (1992) Factors influencing the biocompatibility of insertable silicon microshafts in cerebral cortex. IEEE transactions on bio-medical engineering 39: 635–643. 1601445 10.1109/10.141202

[15] Seymour JP, Kipke DR (2007) Neural probe design for reduced tissue encapsulation in CNS. Biomaterials 28: 3594–3607. 17517431 10.1016/j.biomaterials.2007.03.024

[16] Skousen JL, Merriam SM, Srivannavit O, Perlin G, Wise KD, et al (2011) Reducing surface area while maintaining implant penetrating profile lowers the brain foreign body response to chronically implanted planar silicon microelectrode arrays. Progress in brain research 194: 167–180. 21867802 10.1016/B978-0-444-53815-4.00009-1

[17] He W, Bellamkonda RV (2005) Nanoscale neuro-integrative coatings for neural implants. Biomaterials 26: 2983–2990. 15603793 10.1016/j.biomaterials.2004.08.021

[18] Johansson F, Wallman L, Danielsen N, Schouenborg J, Kanje M (2009) Porous silicon as a potential electrode material in a nerve repair setting: Tissue reactions. Acta biomaterialia 5: 2230–2237. 19285930 10.1016/j.actbio.2009.02.010

[19] Lu Y, Wang D, Li T, Zhao X, Cao Y, et al (2009) Poly(vinyl alcohol)/poly(acrylic acid) hydrogel coatings for improving electrode-neural tissue interface. Biomaterials 30: 4143–4151. 19467702 10.1016/j.biomaterials.2009.04.030

[20] Winslow BD, Christensen MB, Yang WK, Solzbacher F, Tresco PA (2010) A comparison of the tissue response to chronically implanted Parylene-C-coated and uncoated planar silicon microelectrode arrays in rat cortex. Biomaterials 31: 9163–9172. 20561678 10.1016/j.biomaterials.2010.05.050 PMC12327938

[21] Biran R, Martin DC, Tresco PA (2007) The brain tissue response to implanted silicon microelectrode arrays is increased when the device is tethered to the skull. J Biomed Mater Res A 82: 169–178. 17266019 10.1002/jbm.a.31138

[22] Kozai TD, Marzullo TC, Hooi F, Langhals NB, Majewska AK, et al (2010) Reduction of neurovascular damage resulting from microelectrode insertion into the cerebral cortex using in vivo two-photon mapping. Journal of Neural Engineering 7: 046011. 20644246 10.1088/1741-2560/7/4/046011 PMC3164482

[23] Eriksson Linsmeier C, Prinz CN, Pettersson LM, Caroff P, Samuelson L, et al (2009) Nanowire biocompatibility in the brain–looking for a needle in a 3D stack. Nano Lett 9: 4184–4190. 19845389 10.1021/nl902413x

[24] He W, McConnell GC, Bellamkonda RV (2006) Nanoscale laminin coating modulates cortical scarring response around implanted silicon microelectrode arrays. Journal of Neural Engineering 3: 316–326. 17124336 10.1088/1741-2560/3/4/009

[25] Lind G, Linsmeier CE, Thelin J, Schouenborg J (2010) Gelatine-embedded electrodes–a novel biocompatible vehicle allowing implantation of highly flexible microelectrodes. Journal of Neural Engineering 7: 046005. 20551508 10.1088/1741-2560/7/4/046005

[26] Linsmeier CE, Wallman L, Faxius L, Schouenborg J, Bjursten LM, et al (2008) Soft tissue reactions evoked by implanted gallium phosphide. Biomaterials 29: 4598–4604. 18801568 10.1016/j.biomaterials.2008.08.028

[27] Bao F, Chen Y, Dekaban GA, Weaver LC (2004) Early anti-inflammatory treatment reduces lipid peroxidation and protein nitration after spinal cord injury in rats. Journal of neurochemistry 88: 1335–1344. 15009633 10.1046/j.1471-4159.2003.02240.x

[28] Kim S, Bhandari R, Klein M, Negi S, Rieth L, et al (2009) Integrated wireless neural interface based on the Utah electrode array. Biomedical microdevices 11: 453–466. 19067174 10.1007/s10544-008-9251-y

[29] Alonso G (2005) NG2 proteoglycan-expressing cells of the adult rat brain: possible involvement in the formation of glial scar astrocytes following stab wound. Glia 49: 318–338. 15494983 10.1002/glia.20121

[30] Buffo A, Rite I, Tripathi P, Lepier A, Colak D, et al (2008) Origin and progeny of reactive gliosis: A source of multipotent cells in the injured brain. Proceedings of the National Academy of Sciences of the United States of America 105: 3581–3586. 18299565 10.1073/pnas.0709002105 PMC2265175

[31] Komitova M, Serwanski DR, Lu QR, Nishiyama A (2011) NG2 cells are not a major source of reactive astrocytes after neocortical stab wound injury. Glia 59: 800–809. 21351161 10.1002/glia.21152 PMC3560299

[32] Cavanagh JB (1970) The proliferation of astrocytes around a needle wound in the rat brain. Journal of anatomy 106: 471–487. 4912665 PMC1233423

[33] Latov N, Nilaver G, Zimmerman EA, Johnson WG, Silverman AJ, et al (1979) Fibrillary astrocytes proliferate in response to brain injury: a study combining immunoperoxidase technique for glial fibrillary acidic protein and radioautography of tritiated thymidine. Developmental biology 72: 381–384. 389711 10.1016/0012-1606(79)90127-1

[34] Chojnacki AK, Mak GK, Weiss S (2009) Identity crisis for adult periventricular neural stem cells: subventricular zone astrocytes, ependymal cells or both? Nature reviews Neuroscience 10: 153–163. 19153578 10.1038/nrn2571

[35] Levison SW, Goldman JE (1993) Both oligodendrocytes and astrocytes develop from progenitors in the subventricular zone of postnatal rat forebrain. Neuron 10: 201–212. 8439409 10.1016/0896-6273(93)90311-e

[36] Andjelkovic AV, Kerkovich D, Shanley J, Pulliam L, Pachter JS (1999) Expression of binding sites for beta chemokines on human astrocytes. Glia 28: 225–235. 10559781

[37] Bush TG, Puvanachandra N, Horner CH, Polito A, Ostenfeld T, et al (1999) Leukocyte infiltration, neuronal degeneration, and neurite outgrowth after ablation of scar-forming, reactive astrocytes in adult transgenic mice. Neuron 23: 297–308. 10399936 10.1016/s0896-6273(00)80781-3

[38] Faulkner JR, Herrmann JE, Woo MJ, Tansey KE, Doan NB, et al (2004) Reactive astrocytes protect tissue and preserve function after spinal cord injury. The Journal of neuroscience : the official journal of the Society for Neuroscience 24: 2143–2155. 14999065 10.1523/JNEUROSCI.3547-03.2004 PMC6730429

[39] Pekny M, Johansson CB, Eliasson C, Stakeberg J, Wallen A, et al (1999) Abnormal reaction to central nervous system injury in mice lacking glial fibrillary acidic protein and vimentin. The Journal of cell biology 145: 503–514. 10225952 10.1083/jcb.145.3.503 PMC2185074

[40] Pekny M, Nilsson M (2005) Astrocyte activation and reactive gliosis. Glia 50: 427–434. 15846805 10.1002/glia.20207

[41] McKeon RJ, Schreiber RC, Rudge JS, Silver J (1991) Reduction of neurite outgrowth in a model of glial scarring following CNS injury is correlated with the expression of inhibitory molecules on reactive astrocytes. The Journal of neuroscience : the official journal of the Society for Neuroscience 11: 3398–3411. 1719160 10.1523/JNEUROSCI.11-11-03398.1991 PMC6575543

[42] Wilhelmsson U, Li L, Pekna M, Berthold CH, Blom S, et al (2004) Absence of glial fibrillary acidic protein and vimentin prevents hypertrophy of astrocytic processes and improves post-traumatic regeneration. The Journal of neuroscience : the official journal of the Society for Neuroscience 24: 5016–5021. 15163694 10.1523/JNEUROSCI.0820-04.2004 PMC6729371

